# Antifungals in Patients With Extracorporeal Membrane Oxygenation: Clinical Implications

**DOI:** 10.1093/ofid/ofae270

**Published:** 2024-05-08

**Authors:** Lisa Kriegl, Stefan Hatzl, Gernot Schilcher, Ines Zollner-Schwetz, Johannes Boyer, Christina Geiger, Martin Hoenigl, Robert Krause

**Affiliations:** Division of Infectious Diseases, Department of Internal Medicine, Medical University of Graz, Graz, Austria; BioTechMed-Graz, Graz, Austria; BioTechMed-Graz, Graz, Austria; Intensive Care Unit, Department of Internal Medicine, Medical University of Graz, Graz, Austria; Landeskrankenhaus Südsteiermark, Wagna, Austria; Division of Infectious Diseases, Department of Internal Medicine, Medical University of Graz, Graz, Austria; Division of Infectious Diseases, Department of Internal Medicine, Medical University of Graz, Graz, Austria; Division of Infectious Diseases, Department of Internal Medicine, Medical University of Graz, Graz, Austria; Division of Infectious Diseases, Department of Internal Medicine, Medical University of Graz, Graz, Austria; BioTechMed-Graz, Graz, Austria; Division of Infectious Diseases, Department of Internal Medicine, Medical University of Graz, Graz, Austria; BioTechMed-Graz, Graz, Austria

**Keywords:** antifungal prophylaxis, antifungal treatment, extracorporeal membrane oxygenation (ECMO), fungal infections, intensive care

## Abstract

Extracorporeal membrane oxygenation (ECMO) is a life-saving technique used in critical care medicine for patients with severe respiratory or cardiac failure. This review examines the treatment and prophylaxis of fungal infections in ECMO patients, proposing specific regimens based on available data for different antifungals (azoles, echinocandins, amphotericin B/liposomal amphotericin B) and invasive fungal infections. Currently, isavuconazole and posaconazole have the most supported data, while modified dosages of isavuconazole are recommended in ECMO. Echinocandins are preferred for invasive candidiasis. However, choosing echinocandins is challenging due to limited and varied data on concentration loss in the ECMO circuit. Caution is likewise advised when using liposomal amphotericin B due to uncertain concentrations and potential ECMO dysfunction based on scarce data. We further conclude with the importance of further research on the impact of ECMO on antifungal drug concentrations to optimize dosing regimens in critically ill patients.

## Teaser

This review summarizes antifungal use in ECMO patients, exploring specific substances, dosing, TDM and ECMO-related factors from a clinical point of view. It addresses challenges in optimizing drug regimens and offers clinical recommendations for critically ill ECMO patients.

Extracorporeal membrane oxygenation (ECMO) has emerged as a last resort treatment option for patients with severe heart and/or lung failure in critical care medicine. Recently, severe acute respiratory syndrome coronavirus 2 (SARS-CoV-2) causing the coronavirus disease 2019 (COVID-19) pandemic brought intensive care units (ICUs) to the verge of collapse, rescuing patients who would have died under conventional therapy [1].

Despite the potential of ECMO as a bridge to recovery or a bridge to lung/heart transplantation, several complications such as infections, bleeding, blood clot formation, and kidney failure are limiting factors of this approach. Infections associated with ECMO support constitute a significant challenge for infectious disease specialists and intensivists and lead to increased mortality as well as morbidity. The risk of ECMO-associated infections increases with duration of ECMO support [2]. Infections during ECMO therapy range from 8.8% to 64% due to heterogeneous cohorts in the literature [3]. In addition to bacterial infections commonly seen in ECMO-associated infections, potentially life-threatening fungal infections can also pose a risk to these patients. When discussing fungal infections in ECMO patients, it is important to highlight 2 main entities, which may arise due to underlying disease or the use of artificial organ support:

1. invasive pulmonary mold infections (mainly caused by *Aspergillus* spp.), which are frequently observed in severely immunocompromised individuals and in severe forms of viral pneumonia (eg, influenza, SARS-CoV-2); and
2. invasive *Candida* infections, representing a common microorganism in bloodstream infections in ECMO patients [4, 5].

In a recently published study investigating 368 patients with veno-venous ECMO, the incidence of invasive fungal disease (IFD) was 5.5% (5% *Candida* bloodstream infections, 0.5% invasive aspergillosis) [6]. Other reports from patients with ECMO highlighted the risk for *Candida* infections and the challenges in diagnosis and treatment, including blood cultures from in- and outflow lines in addition to venous puncture, and prompt change of the ECMO system accompanied by intravenous antifungal therapy [7, 8]. The requirement of continuous support by the ECMO system delays or even excludes the exchange of the ECMO system in some cases, which is a requirement for successful treatment of fungal bloodstream infection in patients with ECMO [8, 9].

The impact of fungal disease in ECMO patients is further highlighted by in-hospital mortality rates of 82% in patients with ECMO and IFD, compared with 41% in non-IFD patients with ECMO [6].

In recent years, invasive pulmonary aspergillosis (IPA) has been increasingly reported as a serious and potentially lethal complication in patients requiring ICU treatment for severe influenza or COVID-19-associated acute respiratory failure (with or without ECMO). The median incidence of influenza-associated pulmonary aspergillosis (IAPA) and COVID-19-associated pulmonary aspergillosis (CAPA) among mechanically ventilated patients across studies was 10%–20%, strongly depending on the frequency of bronchoscopies, bronchoalveolar lavage (BAL) sampling, and applied methods [10–13]. While CAPA and IAPA frequently occur in patients without any other underlying disease or risk factors for IPA, IPA has also been described as a coinfection in immunocompromised solid-organ transplant recipients with respiratory syncytial virus (RSV), parainfluenza, or adenovirus infections [14].

It is of utmost importance that critically ill patients with ECMO receive appropriate antifungal prophylaxis or treatment, as inadequate dosing can increase the risk of treatment failure and resistance development, and excessive dosing may lead to an increase in adverse effects. To date, limited data exist regarding the effects of ECMO (plus/minus renal replacement therapy [RRT]) on antifungal drug concentrations, despite its increased use in ICUs. In addition to known pathophysiological changes in ICU patients such as peripheral vasodilation, hypoalbuminemia, and renal and/or liver failure, it is necessary to elucidate the influence of the ECMO system itself on altered concentrations of antifungals. Critically ill patients often already exhibit altered pharmacokinetics (PK), such as changes in volume of distribution and clearance (which tend to increase during the early stages of sepsis but to decrease in later stages), renal or liver failure, altered blood pH, hypoalbuminemia, and drug–drug interactions [15]. Application of ECMO and additional extracorporeal treatments (eg, RRT) can further influence these PK changes through factors related to the ECMO and/or the RRT system (eg, priming volumes, technical ECMO factors like material and flow rate, sequestration, removal of hydrophilic drugs by RRT, and others) [16]. Acute kidney injury (AKI) during ECMO is associated with poor outcomes (3.7-fold higher hospital mortality), with almost 50% of patients requiring additional RRT [17]. In critically ill patients receiving RRT, most antifungal agents commonly used to treat IFD do not require dosing adjustments, except for fluconazole, itraconazole, and flucytosine [18]. Estimating changes in antifungal PK in patients receiving both ECMO and RRT support can be challenging. It is not a simple additive effect, as the impacts may also counteract each other and depend on the physicochemical properties of the drug [18]. Primarily, the choice of antifungal agent depends on the indication (either prophylaxis or treatment) as well as confirmed or suspected fungal infection, the patient's condition, potentially altered organ dysfunction, concomitant medication, and other factors. In addition, PK parameters and potentially modified dosing regimens must be considered in patients with ECMO and certain indications for administration of antifungal agents. In a recent review, critical illness–related physiological changes and associated alterations of PK parameters, like volume of distribution and drug clearance as well as protein binding of lipophilic substances and potential sequestration of antifungal drugs within the ECMO circuit, were extensively discussed [15]. However, a comprehensive review of clinically applicable dosing regimens in patients with ECMO is lacking.

Thus, the aim of this review is to contribute data from the recent literature and to derive clinical implications, that is, for the selection of specific antifungal agents, dosing, and dose interval with regard to specific situations (prophylaxis, treatment of candidiasis, aspergillosis, mucormycosis, other specific fungal infections). Based on the available literature, we aim to propose optimized regimens of antifungal agents in patients with ECMO.

## METHODS

We performed a structured literature review by searching PubMed from July 2022 to December 2023 to include recent papers in addition to the literature review truncated in July 2022 and published recently [15]. To put new data into context, some of the already reviewed data are nevertheless included here as deemed necessary by the authors. We additionally performed a temporally unrestricted literature review for simulated or investigated dosing regimens of antifungal agents in patients with ECMO and derived antifungal dosing regimens applicable in “clinical routine” and therefore describing settings of patients treated in ICUs and receiving treatments with ECMO and antifungals. Search terms included “pharmacokinetics AND/OR dosing,” “extracorporeal membrane oxygenation AND/OR ECMO,” AND/OR “antifungal” AND/OR “fluconazole” AND/OR “itraconazole” AND/OR “isavuconazole” AND/OR “posaconazole” AND/OR “voriconazole” AND/OR “anidulafungin” AND/OR “caspofungin” AND/OR “micafungin” AND/OR “amphotericin” AND/OR “rezafungin” AND/OR “fosmanogepix” AND/OR “ibrexafungerp” AND/OR “olorofim.” Articles included case series, case reports, clinical PK studies, in vitro and ex vivo studies, and investigations of the ECMO circuit. We did not include studies in patients younger than 16 years of age, animal studies, or studies in languages other than English. We included recently licensed antifungals and those with potential indications for prophylaxis or treatment of invasive fungal infections [19]. We did not include antifungals with indications exclusively outside the ICU, such as oteseconazole for vulvovaginal candidiasis in outpatient settings.

## ANTIFUNGAL AGENTS

### Fluconazole

Recently, fluconazole plasma concentrations were measured in 8 critically ill patients receiving concomitant ECMO and continuous RRT. Five patients received 800 mg of fluconazole on day 1 (8–10 mg/kg body weight) followed by 400 mg q24 hours (4–5 mg/kg body weight), 2 patients received 400 mg q24 hours (6 mg/kg body weight), and 1 patient received 100 mg q24 hours (1 mg/kg body weight) [16]. Dosing simulations showed that current guidelines (initial loading dose of 12 mg/kg on day 1, followed by 6 mg/kg q24h on day 2 and the following days) achieved a >90% probability of target attainment (PTA; AUC0–24/MIC >100 at days 1 and 7) for a minimal inhibitory concentration (MIC) of *Candida* spp. isolates up to 1 mg/L. None of the tested dosing regimens achieved a 90% PTA for MICs >2 mg/L. Simulated dosing regimens including a loading dose of 18 mg/kg achieved a >90% PTA for an MIC of up to 2 mg/L, regardless of the total body weight. On day 7, only increasing the maintenance dose to 12 mg/kg q24h or reducing the dosing interval to q12h achieved a >90% PTA for an MIC up to 2 mg/L. None of the tested dosing regimens achieved efficacious PTA for MICs >2 mg/L [16].

### Voriconazole

Subtherapeutic concentrations of voriconazole and loss of voriconazole in the ECMO oxygenator have been reported in a recent review [15]. Based on the anticipated loss to the ECMO system, a dose escalation was applied in 2 patients, which surprisingly led to supratherapeutic concentrations (trough levels >10 mg/L, peak 15 mg/L), obviously due to saturation of the ECMO circuit [15, 20]. In another ECMO case report, approximately twice as much as the routine dose was necessary to achieve the target concentration of voriconazole [21]. A recent ex vivo ECMO study demonstrated a loss of 27% in voriconazole concentrations in ECMO samples compared with 19% in non-ECMO controls within 24 hours. The latter represented samples drawn at baseline from the same sampling port just after the ECMO oxygenator, stored for the same period, and subsequently submitted to analysis as the corresponding ECMO samples. Thus, there was obvious adsorption of voriconazole to the ECMO oxygenator within this 24-hour time frame, but to a lesser extent than other antifungals also included in this study (posaconazole and caspofungin, see below) [22]. The results of this ex vivo analysis are in contrast to others, which showed a marked decrease of voriconazole concentrations (up to 80%) in ex vivo setups using other ECMO manufacturers [23]. The abovementioned ex vivo studies investigated samples drawn minutes after drug administration but truncated measurements after 24 hours [22, 23]. Voriconazole concentrations after this time point can therefore not be assessed from these studies. The discrepancy between these results further illustrates the importance of investigating different ECMO circuits and the need for in vivo studies. However, the recently reviewed PK profiles of voriconazole including case reports and ex vivo studies with inconsistent findings indicate that further research is needed before voriconazole can be used in patients with ECMO [15, 20–23].

### Posaconazole

Posaconazole was significantly sequestrated in an ex vivo blood primed ECMO circuit with antifungal drug loss of 63% compared with 11% in non-ECMO controls 24 hours after posaconazole application (*P* < .005) [22]. This diminished concentration within 24 hours is in line with in vivo data of 6 patients showing a slow but constant increase from subtherapeutic concentrations up to the attained target of 1 mg/L within 48 hours, which is considered sufficient for treatment [24]. However, whereas the probability of target attainment for a simulated cohort 48 hours after initiating posaconazole was 98% for 0.7 mg/L (prophylaxis target), it was markedly lower at 59% for 1 mg/L (treatment target). As the majority, but not all, measured trough levels were above the lower limit for treatment, the authors recommended routine dosage but therapeutic drug monitoring (TDM) to guarantee exposure, especially if higher targets are desired [24].

### Isavuconazole

Recently, isavuconazole plasma concentration measurements in 7 critically ill patients with ECMO showed that isavuconazole was not altered by the ECMO oxygenator and that median plasma concentrations were >1 mg/L 24 hours after the first routine loading dose [25]. In 2 recently published cases, the 24-hour areas under the concentration–time curve and trough concentrations of isavuconazole were lower in both patients with ECMO in comparison with previously published data from non-ECMO patients [26]. Another case series including 9 critically ill patients with IFD and ECMO revealed that isavuconazole plasma levels were lower in patients receiving ECMO therapy compared with patients without ECMO [27]. A recent study included 18 critically ill patients, of whom 5 had CAPA, ECMO, and isavuconazole treatment. Although the authors did not find altered isavuconazole exposure in patients with ECMO, increased loading doses were suggested by simulation of trough concentration data derived from the total cohort [28]. To date, only 1 recent study has investigated adapted isavuconazole dose regimes in patients with ECMO [29]. Based on previous PK data, a stepwise increase of the first isavuconazole loading dose from 200 to 300 mg and finally 400 mg was investigated and resulted in a substantial increase of the median isavuconazole plasma concentrations, achieving ≥1 mg/L immediately and remaining constant after this first dose [29]. Isavuconazonium sulfate is the prodrug of isavuconazole, where 200 mg of isavuconazole is equivalent to 372 mg of isavuconazonium. Due to the small sample size (n = 5 with a 400-mg first dose), a potential impact of increased isavuconazole loading dosages on clinical outcomes and survival could not be evaluated. While animal models of invasive aspergillosis outline that a delay of treatment initiation by 24 hours may already impact efficacy, more studies are needed to evaluate the impact of low isavuconazole concentrations within the first 24 hours on patient outcomes [29, 30]. In terms of potential toxicity caused by higher loading doses, neither during the loading phase nor at steady state did the plasma concentrations of isavuconazole reach or exceed the threshold of 5 mg/L, which is considered the surrogate toxic threshold [29, 31]. Nevertheless, due to the heterogeneity of critically ill patients treated with ECMO, further studies investigating isavuconazole dosage, TDM, and outcome parameters (treatment success, adverse events) are necessary in patients with ECMO. The methods and time points of azole measurements applied in the dose escalation study can, however, serve as a model for future in vivo studies.

### Caspofungin

Various data from patients with ECMO receiving caspofungin have been reported [18, 20]. The abovementioned and recently published ex vivo ECMO study demonstrated a loss of caspofungin in ECMO and non-ECMO samples [22]. Ex vivo caspofungin was obviously sequestrated in the blood primed ECMO circuit with antifungal drug loss of 80% compared with 61% in non-ECMO controls 24 hours after caspofungin application, although the difference was not significant [22]. Although the loss of caspofungin concentration did not significantly differ between the ECMO and non-ECMO samples, a “first-pass effect” of the ECMO oxygenator cannot be excluded based on the ex vivo ECMO setup. This first-pass effect refers to drug sequestration within the ECMO system, resulting in lower concentration of the specific drug. Consequently, the clinical implication might be that certain antifungal agents may need higher loading doses to achieve therapeutic levels. After administration of caspofungin at baseline, time point samples were drawn from the sampling port located beyond the ECMO oxygenator and stored in a water bath at 37°C with tubes removed at the same time points as the ECMO test samples to demonstrate the natural degradation of the antifungal drug over time. Thus, natural drug degradation, metabolism via plasma peptidases, and adsorption to the ECMO tubes or sampling tube were also discussed as possible underlying mechanisms for the unexpected loss of caspofungin concentration in the control samples [22]. Whereas the loss of caspofungin within the ECMO circuit was in line with previous studies (ie, 44% lower concentration compared with controls in another study), there are heterogeneous results ranging from 1% to 15% in previously investigated control samples [32, 33].

### Micafungin

Lower concentrations of micafungin in ex vivo ECMO circuits were shown, with 33% loss compared with 1% in controls 24 hours after micafungin administration [32, 33]. In 12 patients, micafungin was not extracted by the ECMO oxygenator on day 1 and day 4 [34], but the micafungin concentration was reported to be reduced in another study in patients with ECMO by 23% [15]. An increased dosage of 150 mg of micafungin was successful in a patient with *Candida glabrata* fungemia receiving veno-venous ECMO and continuous RRT [35].

### Anidulafungin

One case report is mentioned in the literature with unchanged anidulafungin PK profiles in a patient treated with veno-venous ECMO [36].

### Amphotericin B and Liposomal Amphotericin B

Amphotericin B is reported to be unaffected by ECMO, whereas contradictory findings were shown for liposomal amphotericin B [15, 18], with unchanged or diminished concentrations of liposomal amphotericin B [37]. In addition, in 1 case report ECMO occlusion complicated the treatment of a patient suffering from blastomycosis [38]. In another case report, the applied dosage of liposomal amphotericin B was 6 mg/kg body weight for treatment of invasive trachea-pulmonary aspergillosis in a patient with ECMO, but no concentrations of the antifungal agent were measured [39].

### Other Antifungals

At present, there are no available data on the use of itraconazole, flucytosine, rezafungin, fosmanogepix, and ibrexafungerp in patients with ECMO. Recently, 1 patient with necrotizing pneumonia due to *Microascus melanosporus* was treated with olorofim and underwent ECMO, but no olorofim concentrations were measured [40].

## ECMO-RELATED FACTORS POTENTIALLY INFLUENCING ANTIFUNGAL CONCENTRATIONS

**Figure 1.**
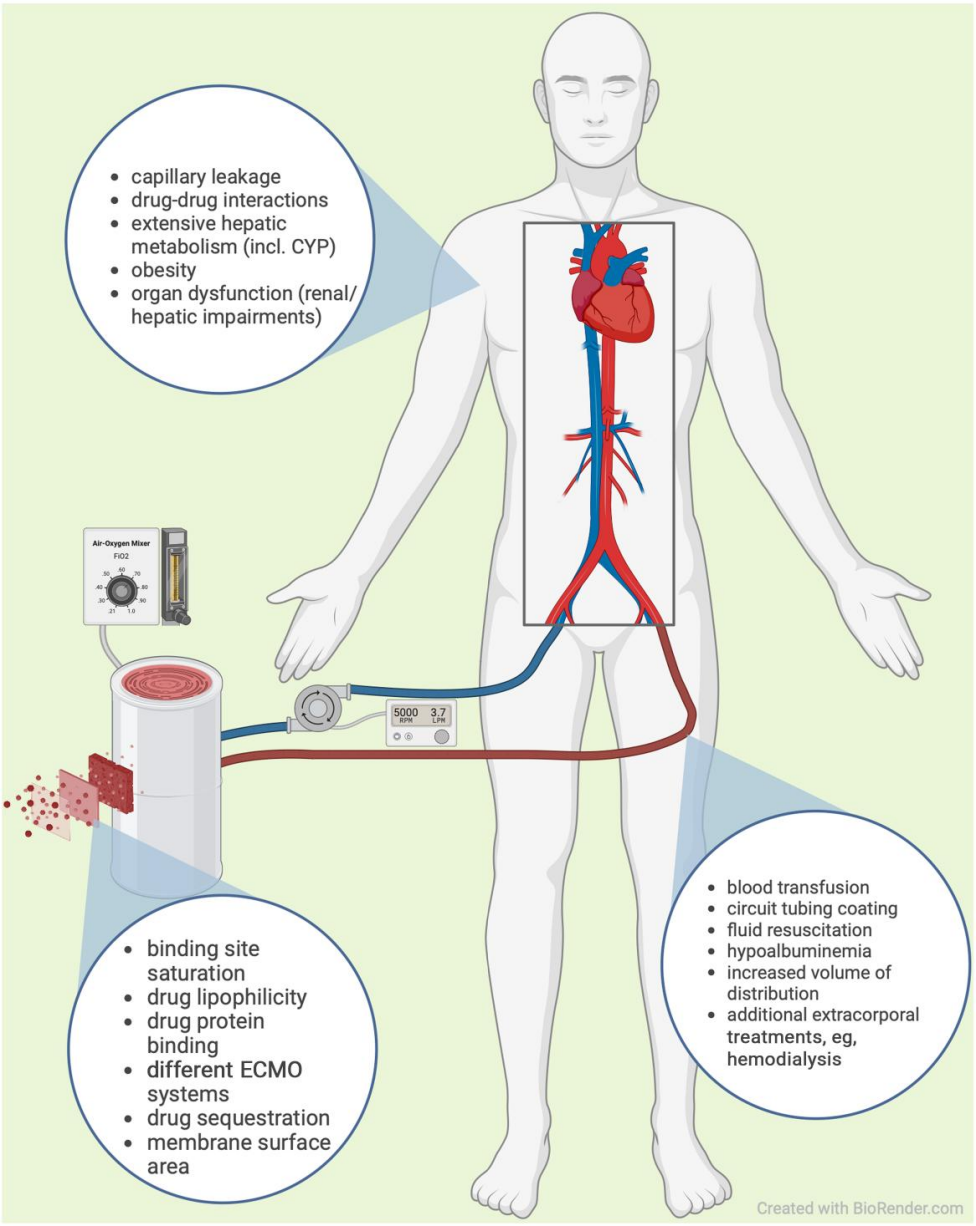
ECMO-related factors potentially influencing antifungal concentrations (attached). Created with Biorender.com. Abbreviations: CYP, Cytochrome P; ECMO, extracorporeal membrane oxygenation.

A variety of ECMO-related factors might contribute to variabilities in antifungal drug concentrations, although most of the data addressing different parts of the ECMO systems were obtained by investigation of nonantifungal drugs (Figure 1). These factors include materials of oxygenators, type of pump (roller pumps or centrifugal pumps) [41], the size/surface area of the oxygenator [42], and coatings of ECMO tubing materials [43]. As mentioned above, drug sequestration by the ECMO circuit was described for voriconazole in ex vivo models and case reports [22, 23]. By investigating pre- and postoxygenator isavuconazole levels, recent data demonstrated that the ECMO system [25] obviously does not contribute to isavuconazole concentration alterations [25]. Thus, the influence of technical ECMO factors might vary and should be further investigated.

## PROPOSED SELECTION AND DOSAGE OF ANTIFUNGAL AGENTS IN SPECIFIC INDICATIONS

Below we propose selected antifungal agents and dosage regimens for patients with ECMO in specific clinical indications. In contrast to non-ECMO patients, it is particularly challenging to select echinocandins due to scarce and heterogeneous data regarding loss of concentration in the ECMO circuit, mainly from ex vivo studies and some case reports. The safety profile of echinocandins might allow calculation of potentially missing dosages based on ex vivo models, but these dose modifications nevertheless need to be investigated in vivo prospectively. Dose modifications of azoles as well as liposomal amphotericin B must be investigated in vivo before administration in clinical routine due to potential side effects. Previously, dose adaptions based on theoretical considerations resulted in toxic voriconazole concentrations and should therefore be avoided [20]. TDM is recommended for azoles and for echinocandins in patients with ECMO [24, 25, 29, 44–46]. Exact time points and frequency of TDM sampling in patients with ECMO and specific antifungals are not available in the literature. In the meantime, time points are inferred from non-ECMO patients (Table 1) and need further investigation. While application of a personalized TDM approach can assist with reaching and maintaining therapeutic levels, reducing the risk of toxicity, and improving treatment outcomes, we suggest conducting these practices preferably within the framework of clinical studies. This procedure allows the collection of valuable data on drug pharmacokinetics and pharmacodynamics, which in turn will contribute to the development of effective antifungal strategies in the future.

**Table 1.** Suggested Dosing and TDM Recommendations for Antifungals in Patients With ECMO

Antifungals	Dosing in ECMO	TDM	Suggested Timing on First TDM Sample^a^	Target Serum Concentrations
Anidulafungin	200 mg IV day 1 q24h, followed by 100 mg IV q24h	Should be considered^b^	Not defined in ECMO patients	-
Micafungin	150 mg IV daily	Should be considered^b^	Not defined in ECMO patients	-
Caspofungin	100 mg IV day 1 q24h, followed by 70 mg (<80 kg body weight) to 100 mg IV (≥80 kg body weight) daily	Should be considered^b^	Not defined in ECMO patients	-
LAmB	5–10 mg/kg body weight depending on indications	No clear recommendation	-	-
Voriconazole	2 × 6 mg/kg body weight IV day 1, followed by 2 × 4 mg/kg body weight IV	Recommended	24–48 h after first dose (loading dose) After 5–7 d	C_min_ 1–5.5 mg/L
Posaconazole	2 × 300 mg IV day 1, followed by 1 × 300 mg IV	Recommended	24–48 h after first dose (loading dose) After 5–7 d	C_min_ >0.7 mg/L for prophylaxis C_min_ >1 mg/L for treatment
Isavuconazole	400 mg IV as the first dose, after 8 h 200 mg IV q 8 h for 5 doses, followed by 200 mg q24h	Suggested	24–48 h after first dose (loading dose) After 3 d (steady state)	C_min_ >1 mg/L
Fluconazole	12 mg/kg	Suggested	5 d after first dose	C_min_ >12 mg/L

Abbreviations: ECMO, extracorporeal membrane oxygenation; IV, intravenous; TDM, therapeutic drug monitoring.

^a^Reference for exact sampling time points in ECMO patients is not available. Recommendations for time points are inferred from non-ECMO patients. Consider additional determination of concentrations: After a change in dose, introduction or discontinuation of drugs with significant interaction potential, disease progression, concern for toxicity, after exchange of ECMO system, after introduction of additional extracorporeal treatments to ECMO (eg, renal replacement therapy).

^b^[45, 47].

### Antifungal Prophylaxis

Based on the indications for adult antifungal prophylaxis, we propose fluconazole or posaconazole in routinely applied dosages (ie, fluconazole 1 × 10–12 mg/kg body weight followed by at least 6 mg/kg body weight; posaconazole 2 × 300 mg on day 1 followed by 1 × 300 mg per day on day 2 and the following days). The implications of delayed attainment of posaconazole target concentration in patients with ECMO [24] are uncertain [44, 46]. Isavuconazole might be another option for prophylaxis, as the target concentration in a routinely applied dosage is faster attained compared with posaconazole in patients with ECMO. As described above, increasing the isavuconazole starting dose to 400 mg followed by the standard 200 mg q8-hours for 5 doses, then 200 mg daily, results in immediate and continuous concentrations >1 mg/L [29]. We do not recommend micafungin in a standard prophylactic dosage due to decreased concentrations in patients with ECMO. If an echinocandin is being considered for prophylactic purposes, anidulafungin in a standard dose or caspofungin or micafungin might be options (micafungin 150 mg or caspofungin 100 mg followed by 70 mg (<80 kg of body weight) to 100 mg (≥80 kg of body weight) [15, 20, 32, 37].

### Antifungal Treatment

Based on the PK data and simulated or investigated dosage regimens of antifungals in adult patients with ECMO, the following treatment strategies can be proposed (Table 1).

- Echinocandins are recommended as first-line antifungal agents for invasive candidiasis including candidemia [48–50]. For treatment of invasive candidiasis in patients with ECMO, we propose caspofungin or anidulafungin or micafungin. We propose unchanged dosages of anidulafungin (ie, 200 mg on day 1 q24h, followed by 100 mg q24h on the following days); micafungin 150 mg daily or caspofungin 70 mg (<80 kg of body weight) to 100 mg (≥80 kg of body weight) daily after a loading dose of 100 mg based on ex vivo data and case reports [15, 20, 32, 37].
- In candidemia plus endophthalmitis, antifungal treatment and surgical intervention should be decided jointly by an ophthalmologist and an infectious diseases physician [48]. In patients with ECMO suffering from candidemia plus endophthalmitis, we propose an echinocandin as mentioned above for treatment of candidemia plus fluconazole (12 mg/kg daily) or isavuconazole (400 mg isavuconazole as the first dose followed by 200 mg q8 hours for the next 5 doses [resulting in 6 doses within 48 hours] and by a maintenance dose of 200 mg q24h) [16, 29] for treatment of the eye infection. Although data are limited for isavuconazole, uncertain concentrations are achieved with voriconazole in patients with ECMO, and posaconazole target concentrations are delayed up to 48 hours. Intravitreal voriconazole or amphotericin B [51] is not considered to be affected by ECMO.
- For treatment of invasive aspergillosis in patients with ECMO, we propose isavuconazole with the modified loading dosages mentioned above [29].
- For treatment of mucormycosis in patients with ECMO, we propose a combination of liposomal amphotericin B 5–10 mg/kg body weight plus isavuconazole, with an increased loading dose as mentioned above [29, 52]. We propose this combination based on the recent first-line recommendation for liposomal amphotericin B in mucormycosis, although amphotericin B concentrations are uncertain in patients with ECMO. In addition, based on a case report of ECMO oxygenator occlusion during liposomal amphotericin B administration [38], thorough observation of transmembrane pressure must be applied as occlusion might therefore be detected even before clots are visible by inspection of the oxygenator, followed by immediate cessation of liposomal amphotericin B treatment in cases of suspected ECMO dysfunction.
- For treatment of endemic fungal infections requiring ECMO, we propose, based on specific indications [53], fluconazole in standard dosages or isavuconazole with the modified increased loading dose [29]. In case of indications for liposomal amphotericin B, we propose administration of at least 5 mg/kg body weight per day and thorough observation of ECMO for complications, as described above.

These recommendations apply to adults. Currently, other antifungal agents than the ones discussed above cannot be recommended in patients with ECMO due to missing data or decreased concentration without clear compensatory dosing regimens. New antifungal agents with potential indications in patients with ECMO should be investigated with regard to pharmacokinetics and potential alterations by the ECMO system. Bearing in mind the heterogeneity of critically ill patients treated with ECMO and the variability in applied materials, further studies addressing dosages of antifungals, TDM, and related outcomes in patients with ECMO are necessary.
